# Platelet Priming and Activation in Naturally Occurring Thermal Burn Injuries and Wildfire Smoke Exposure Is Associated With Intracardiac Thrombosis and Spontaneous Echocardiographic Contrast in Feline Survivors

**DOI:** 10.3389/fvets.2022.892377

**Published:** 2022-07-14

**Authors:** Avalene W. K. Tan, Ronald H. L. Li, Yu Ueda, Joshua A. Stern, Mehrab Hussain, Satoshi Haginoya, Ashely N. Sharpe, Catherine T. Gunther-Harrington, Steven E. Epstein, Nghi Nguyen

**Affiliations:** ^1^William R. Pritchard Veterinary Medical Teaching Hospital, School of Veterinary Medicine, University of California, Davis, Davis, CA, United States; ^2^Department of Surgical and Radiological Sciences, School of Veterinary Medicine, University of California, Davis, Davis, CA, United States; ^3^Department of Clinical Sciences, College of Veterinary Medicine, North Carolina State University, Raleigh, NC, United States; ^4^Department of Medicine and Epidemiology, School of Veterinary Medicine, University of California, Davis, Davis, CA, United States

**Keywords:** hypercoagulability, primary hemostasis, particulate matter <2.5 μm (PM) 2.5, hypertrophic cardiomyopathy (HCM), thromboembolism

## Abstract

Wildfires pose a major health risk for humans, wildlife, and domestic animals. We previously discovered pathophysiologic parallels between domestic cats with naturally occurring smoke inhalation and thermal burn injuries and human beings with similar injuries; these were characterized by transient myocardial thickening, cardiac troponin I elevation and formation of intracardiac thrombosis. While the underlying mechanisms remain unclear, results from murine models suggest that platelet priming and activation may contribute to a global hypercoagulable state and thrombosis. Herein, we evaluated and compared the degree of platelet activation, platelet response to physiologic agonists and levels of platelet-derived microvesicles (PDMV) in 29 cats with naturally occurring wildfire thermal injuries (WF), 21 clinically healthy cats with subclinical hypertrophic cardiomyopathy (HCM) and 11 healthy cats without HCM (CC). We also quantified and compared circulating PDMVs in WF cats to CC cats. In addition, we examined the association between thrombotic events, severity of burn injuries, myocardial changes, and the degree of platelet activation in cats exposed to wildfires. Flow cytometric detection of platelet surface P-selectin expression showed that WF cats had increased platelet response to adenosine diphosphate (ADP) and thrombin compared to the two control groups indicating the presence of primed platelets in circulation. In addition, cats in the WF group had increased circulating levels of PDMV, characterized by increased phosphatidylserine on the external leaflet. Cats in the WF group with documented intracardiac thrombosis had elevated platelet activation and platelet priming in the presence of ADP. While high dose arachidonic acid (AA) mostly resulted in platelet inhibition, persistent response to AA was noted among cats in the WF group with intracardiac thrombosis. Univariate and multiple logistic regression analyses demonstrated that increased platelet response to AA was independently associated with thrombotic events. This is the first study reporting the significant association between platelet priming and intracardiac thrombosis in domestic cats with naturally occurring wildfire-related injuries and smoke inhalation. Further studies are required to delineate additional mechanisms between inflammation and thrombosis, especially regarding platelet primers and the cyclooxygenase pathway.

**One Sentence Summary:**

Platelet activation and shedding of platelet-derived microvesicles due to platelet priming is present following naturally occurring wildfire smoke exposure and thermal burn injuries in a population of domestic cats.

## Introduction

The most recent wildfires in Northern California (2018 California Camp Fire, 2020 Californian Fires), Brazil (Amazon Fires 2019) and Australia (2019/2020 Australian bushfires) have been the most destructive in modern history, injuring thousands and claiming the lives of billions of animals. As the intensity and frequency of wildfire disasters increase due to climate change, wildfires pose a major health risk for humans, wildlife and domestic animals. An observational study in domestic cats during two recent periods of Californian wildfires (Tubbs Fire 2017, Camp Fire 2018) documented the clinical impacts of wildfire injuries on the cardiovascular system (1). Echocardiography not only documented the presence of transient myocardial thickening (MT) but also spontaneous echocardiographic contrast and intracardiac thrombosis (SEC ± T), suggestive of a hypercoagulable state following wildfire exposure. While human studies have also demonstrated positive associations between wildfire-related injuries like smoke inhalation and thermal burns and increased risk of cardiovascular events, such as myocardial infarction and ischemic diseases (2–5), the underlying mechanisms of these observations are not well understood.

People and animals rescued from wildfires often succumb to thermal burn injuries and smoke inhalation, both of which can result in severe derangements in hemostasis. Observational studies in humans and murine models of burn injuries describe an initial hypercoagulable state despite the presence of thrombocytopenia (6–9). This finding suggests that systemic platelet activation, which likely precedes platelet consumption, plays a crucial role in mediating hypercoagulability as a result of burn injuries (7, 10, 11). Although the exact mechanisms of increased platelet activation in these patients are unknown, endothelial stress and injury during inflammation may facilitate platelet rolling and activation, and subsequent platelet-immune cell interactions via toll-like receptors (TLR) fueling further inflammation (12–15). This highlights the important role that platelets play in linking inflammation with the coagulation system. Platelet activation also triggers the shedding of platelet-derived microvesicles (PDMV). As mediators of hemostasis and inflammation, overzealous amounts of circulating PDMV may further aggravate an already hypercoagulable state (11, 13, 16). Inhalation of ambient particulate matter and noxious gases generated during wildfires may also directly activate circulating platelets. *In vitro* and *in vivo* animal models demonstrate that inhalation of particulate matter can initiate platelet priming, an exaggerated response to physiologic agonists, resulting in systemic platelet activation and elevation of circulating PDMV (17). To date, cardiovascular events and thrombosis as a result of increased platelet priming and shedding of PDMV in animals with naturally occurring wildfire injuries and smoke inhalation have never been demonstrated.

Cats with hypertrophic cardiomyopathy (HCM), the most common primary cardiomyopathy, are at high risk of thromboembolic complications such as sudden death and arterial thromboembolism. Although the exact mechanisms of thrombosis secondary to HCM are unclear, increased platelet priming, endothelial dysfunction and blood flow stasis due to left atrial (LA) dysfunction are proposed to play a key role in contributing to a hypercoagulable state (18–20). Since cats with wildfire-related injuries were previously shown to have transient cardiomyopathies (1), it is plausible that cardiovascular complications and thrombosis noted in these cats may be secondary to similar pathophysiologic mechanisms in HCM cats. A better understanding of the mechanisms behind wildfire related thrombosis will aid in developing more effective thromboprophylaxis and antithrombotic therapies in animals with naturally occurring wildfire injuries.

Our main objective was to evaluate and compare platelet activation and platelet response to physiologic agonists in cats with naturally occurring wildfire injuries, and clinically healthy cats with or without HCM. Circulating PDMV in healthy controls cats and cats with wildfire-related injuries were also quantified and compared. Through this observational study, we examined the association between thrombotic events, severity of burn injuries, myocardial changes, and the degree of platelet activation and priming in cats exposed to wildfire. We hypothesize that WF cats, similar to HCM cats, would have increased response to physiologic agonists due to platelet priming. In addition, this potentiated platelet response to agonists would be associated with thrombotic events.

## Materials and Methods

### Animals and Study Design

The study was a prospective cohort study. Clinical cats were enrolled in accordance with the Institutional Animal Care and Use Committee (IACUC) at the University of California, Davis. Those exposed to wildfires were treated according to the best veterinary standard of care and those that served as controls were enrolled as part of IACUC protocols #20095 and #21037. All experimental protocols were approved by the IACUC of the University of California, Davis and all methods were carried out in compliance with the ARRIVE guidelines (https://arriveguidelines.org/) and regulations for care and use of laboratory animals. Informed owner consent was obtained prior to enrolling all client-owned cats in this study.

### Clinical Cats With Thermal and Smoke Inhalation Injury

Twenty-nine cats presented to the Veterinary Medical Teaching Hospital of the University of California, Davis for treatment of thermal burn injury and smoke inhalation due to the 2018 California Camp Fire were enrolled in this arm of the study. The severity of thermal burn injuries was retrospectively scored as 1 (mild), 2 (moderate) or 3 (severe) based on clinical records and photographic documentation as previously described (1). Briefly, mild burn injury consisted of superficial burns that required minimal to no bandaging. Moderate burn injuries consisted of second degree burns that required daily bandage changes while severe burns were classified as burns involving the head, and all 4 limbs with or without bone exposure. Medical treatment of wildfire-exposed cats was left to the clinicians' discretion and detailed as previously reported (1). All cats with moderate to severe burns were treated with topical silver sulfadiazine and daily bandage changes with or without debridement under sedation. All samples were collected within 7 days after presentation. Aspirin, clopidogrel or a combination of both were commenced in majority of the cats after the results of platelet function analysis from this study (**Table 2**). Age was difficult to determine among cats in the WF group, but all cats were considered adult (≥1 year of age) at the time of presentation.

### Healthy Cats With or Without Subclinical Hypertrophic Cardiomyopathy

Thirty-two student, staff or client-owned cats ≥1 year of age were recruited during the period of October 2018 to June 2019. All cats received a physical examination, complete blood count (Abaxis, Union City, CA), total thyroxine (Abaxis, Union City, CA) and blood pressure measurement via Doppler with sphygmomanometery prior to echocardiography. Cats were excluded if they had a previous history of CHF, uncooperative temperament, apparent systemic diseases including abnormal findings in physical examination, hematology, systemic hypertension (systolic blood pressure >160 mmHg) or hyperthyroidism (>4.8 μg/dl). All cats were screened and confirmed for the presence of HCM with echocardiography and assigned to the HCM or CC groups. Oral administration of gabapentin (50–100 mg) prior to enrollment was permitted.

### Echocardiography

Transthoracic echocardiographic examinations were performed by board-certified veterinary cardiologists (JAS, CGH) or cardiology trainees (ANS, YU) under direct supervision of a board-certified cardiologist. Standard imaging planes were used with the patient in right and left lateral recumbency. One of two machines were used with a 12–4 mHz sector array transducer (Philips iE33 Ultrasound, Philips Health Care, Andover, MA). All measurements were evaluated by the investigators using commercially available software (Syngo Dynamic Workspace 10.0.01, Siemens Medical Solutions, Malvern, PA).

Diagnosis of HCM was made based on identification of idiopathic left ventricular (LV) hypertrophy characterized by regional or global end-diastolic wall thickening ≥6 mm, determined by 2-dimenional or M-mode echocardiography while avoiding inclusion of moderator band insertion sites (21). Myocardial thickening (MT) in wildfire cats was also defined as a diagnostic wall thickness ≥6 mm. LA enlargement was measured in the right parasternal short axis basilar view and defined as an LA/Ao of ≥1.6. The left auricular flow velocity (LAuV) was measured in the oblique left apical parasternal long axis view with pulsed wave Doppler sample volume at the entrance to the left auricle (22). A LAuV of >47 cm/s was considered normal (22). M-mode was used to acquire measurements of LV internal dimension at end-diastole (LVIDd) and LV internal dimension at end-systole (LVIDs) and fractional shortening (FS%) was calculated using the equation:


$$\begin{array}{l} \text{FS}\;(\%)=\frac{(\text{LVIDd}-\text{LVIDs})\;}{\text{LVIDd}}\;\times 100. \end{array}$$

CHF in the WF group was diagnosed by a combination of clinical signs, echocardiographic findings of LA enlargement with the presence of pericardial and/or pleural effusion and radiographic findings consistent of cardiogenic pulmonary edema.

### Blood Sample Collection

Blood was collected from the jugular or medial saphenous vein with a 22G or 23G butterfly catheter and divided among lithium-heparin tubes and 3.2% trisodium citrate tubes immediately after collection. Cats were gently restrained to minimize the effect of stress on platelet activation. Blood collection was aborted if they were noted to be too stressed prior to or during blood collection. Only samples collected by a single clean venipuncture were used in this study. Following enrollment, a complete blood count was performed in all cats. Citrated blood samples were processed within 60–90 min after collection.

### Generation of Platelet Rich Plasma

Citrated blood was transferred to polypropylene tubes and rested for 30 min at 37°C to facilitate red cell sedimentation. The samples were then centrifuged at room temperature at 200 × g for 5 min, no brakes. Platelet rich plasma (PRP) was then separated and rested for an additional 30 min at 37°C prior to analysis. Swirling characteristic of the PRP was noted to ensure that platelets maintained their discoid shape. A platelet count of PRP was obtained with an automated blood cell analyzer (HM5, Abaxis, Union City, CA) and confirmed by blood smear evaluation when indicated.

### Detection of Platelet P-Selectin by Flow Cytometry

PRP was diluted with Tyrodes HEPES buffer (pH 7.2, 5 mM dextrose, without divalent cations) to a final concentration of 1 × 10^7^ platelets/ml. Platelets were either unstimulated (resting) or activated in the presence of 20 μM ADP (Sigma-Aldrich, St. Louis, MO), or 0.01 U/ml bovine α-thrombin (Haematologic Technologies, Essex Junction, VT) for 15 min at 37°C. Platelet P-selectin was detected by fluorescein isothiocyanate-conjugated rat anti-mouse monoclonal antibody to CD62P (1:200, clone: RB40.34, BD Pharmingen, San Jose, CA). Platelet integrin-β3 was identified using allophycocyanin-conjugated mouse anti-human monoclonal antibodies to CD61 (1:500, Clone: VI-PL2, eBioscience, San Diego, CA). Both antibodies were previously shown to cross-react in cats (19, 20). Samples were subsequently incubated in the dark at 37°C for 45 min before fixation with 1% paraformaldehyde at room temperature for 30 min. Samples were then analyzed using a 5-color flow cytometer (FC500, Beckmann Coulter, Miami, FL).

**Figure 1:**
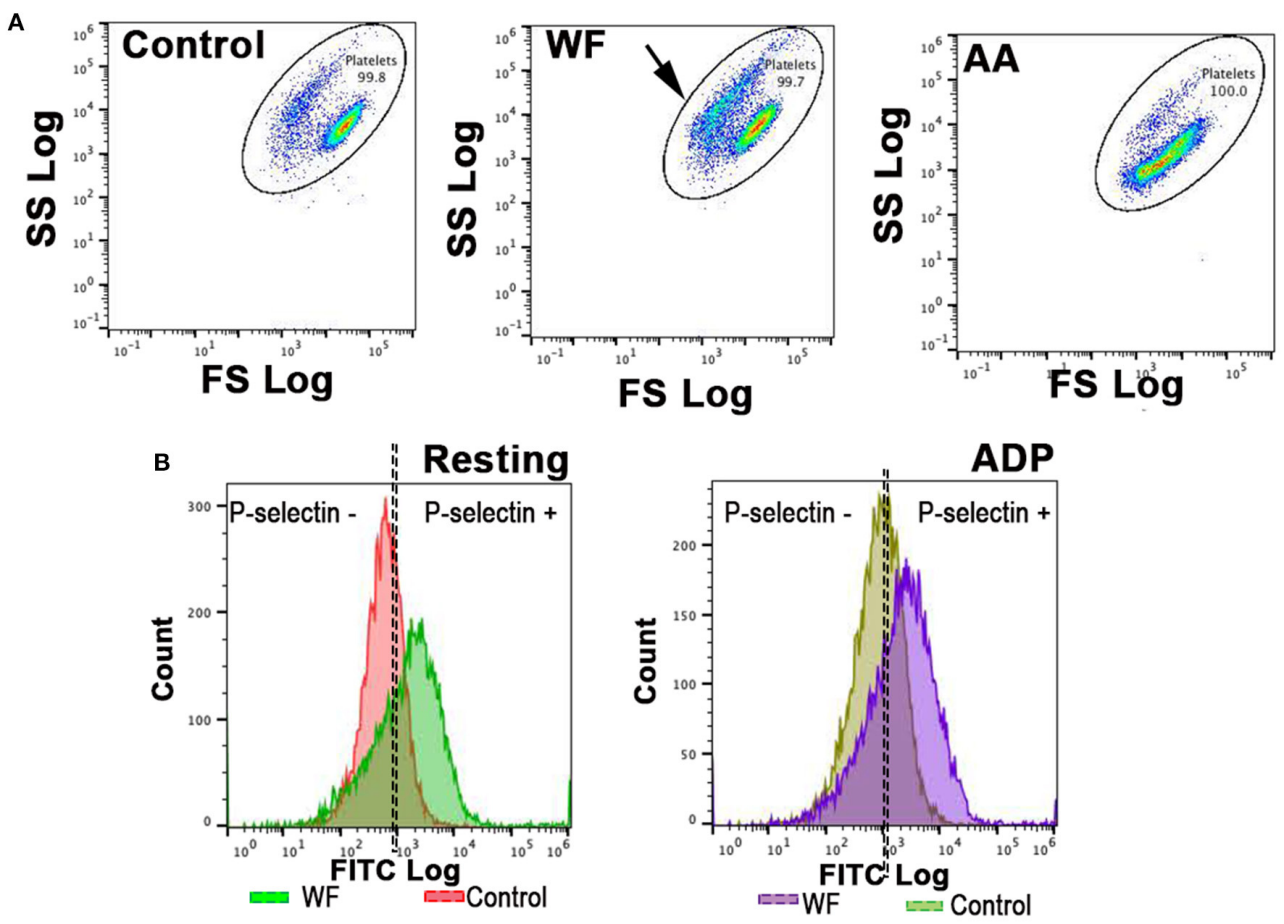
Representative scatter plot diagrams and histograms of flow cytometric analysis of platelets in a cat with wildfire (WF) related injuries and 1 healthy control cat. **(A,B)** Platelets were identified by forward (FS) and side scatter (SS) properties. Note the shift in scatter properties (arrows) in platelets from a cat in WF group compared to those in the healthy control indicating shape change and degranulation. Arachidonic acid (AA)-treated (1 μM) platelets in a WF group cat resulted in a shift in scatter properties indicative of intact granules and size. **(B)** Representative histograms illustrating the number of platelets expressing P-selectin in the absence (resting) or presence of 20 μM ADP in a WF cat and healthy control. Note the upregulation in P-selectin in either resting or ADP-activated platelets in the WF cat compared to healthy control.

Platelets were identified by CD61-positive events as well as forward- and side-scatter characteristics using 0.9 and 3.0 μm calibration beads as previously described (23). Gating was established using fluorescence minus one controls consisting of unstimulated or activated platelets labeled with either one of the antibodies (Figures 1A,B). Fluorescence compensation was applied using anti-mouse isotype controls conjugated to matched fluorophores (BD Biosciences, San Diego, CA) in identical experimental conditions for calculations of compensation matrixes. Flow cytometry data were analyzed using commercially available software (Flowjo, TreeStar Inc, Ashland, OR). Surface P-selectin expression on platelets was quantified as percentage of CD62P-positive events out of 10,000 platelets or median fluorescence intensity (MFI), an indicator of protein density.

In WF cats, platelet activation in response to 1 μM AA (Roche, Basel, Switzerland) was assessed by treating PRP with 1 μM AA for 15 min at 37°C.

### Flow Cytometric Analysis of Platelet Phosphorylation of Vasodilator-Stimulated Phosphoprotein

PRP from 5 healthy cats without HCM was generated and standardized to 2 × 10^7^/ml. Phosphorylation of platelet vasodilator-stimulated phosphoprotein (P-VASP) was measured as previously described by flow cytometry (24). Briefly, platelets were unstimulated or treated with 1 μM AA (Roche, Basel, Switzerland). PGE_1_-treated (10 μM) platelets served as positive control. Platelets were then fixed, permeabilized, washed and labeled with mouse polyclonal antibody conjugated to fluorescein isothiocyanate (5 μg/ml, ALX-804-240F-C100, Enzo Life Sciences, Farmingdale, NY) before analysis on a 5-color flow cytometer (FC500, Beckmann Coulter, Miami, FL).

### Detection of Platelet-Derived Microvesicles by Flow Cytometry

Platelet poor plasma (PPP) from 28 WF and eight cats from the CC group was generated from PRP by further centrifugation at 5,000 × g for 15 min. Supernatant was flash frozen in liquid nitrogen and stored at −80°C until further analysis. PPP was thawed at room temperature and only the top 90% of PPP was used for analysis. Positive controls were generated by treating PRP (1 × 10^7^/ml) from healthy controls with 2 mM calcium chloride (CaCl_2_) over a 60-min interval (1 mM CaCl_2_ every 30 min) and 2.5 μM A23187 (Millipore Sigma, Burlington, MA) for 15 min at 37°C (Figure 6C). To detect PDMV, PPP was incubated in the dark at room temperature for 30 min with phycoerythrin-conjugated monoclonal mouse IgG1 antibodies to CD61 (1:10, Clone: VI-PL2, eBioscience, San Diego, CA) and fluorescein isothiocyanate-conjugated Annexin-V (1:32), diluted in binding buffer [pH 7.4, 100 mM HEPES, 140 mM NaCl, 25 mM CaCl_2_ (BD Biosciences, San Jose, CA)]. Prior to analysis by flow cytometry (FC-500, Beckman Coulter, Brea, CA), samples were further diluted in binding buffer (1:10) and agitated on a vortex for 5 s. No platelet agonists were added to the samples prior to flow cytometry analysis. Gating to quantify PDMV were set by CD61-positive events and Annexin-V positive scatter characteristics using 0.5μm and 3.0μm calibration beads (Megamix, Biocytex) as previously described (Figure 6) (20). PDMV was quantified as a percentage of CD61- and Annexin-V positive events and also by concentration of PDMV (number/μl of PPP). Flow cytometry data were analyzed by commercially available software (Beckman Coulter, Brea, CA).

### Statistics

Assuming biologic variability of 20%, calculations from our preliminary data and standard deviation of data gathered from the 29 WF cats showed that a sample size of at least eight normal cats and 20 HCM cats would be required to demonstrate significant changes with an alpha priori of 0.05 and a power of 80%. Data was assessed for normality with Shapiro–Wilk normality test. Categorical data between groups were compared using a Fisher's exact test or Chi-square analysis. Linear mixed-effect models were used in analysis to further examine the effects between fixed effects of study groups (CC vs. HCM vs. WF), platelet response to agonists (ADP and thrombin), and their interaction, with a random effect of individual cat, nested within the group to account for the assumption of independence. *Post-hoc* pairwise comparisons of means were performed by Tukey's test. The normality of residuals was tested based on Q–Q plot. Homoscedasticity was evaluated using the Breusch–Pagan/Cook–Weisberg test. Unpaired continuous data were analyzed using student *t*-test for normally distributed data or Mann–Whitney test for non-parametric data. Paired continuous data were analyzed using paired *t*-test or Wilcoxan test as appropriate. Spearman's rank correlation analysis was performed between resting and activated platelets. Univariate and multiple logistic regression analyses were performed to predict the development of clot formation and survival by burn severity (1, 2 or 3), platelet count, neutrophil count and degree of platelet activation (P-selectin MFI) and the magnitude of response to ADP, AA and thrombin based on MFI fold change (log_10_ MFI _activated_ – log_10_ MFI _rest_) on a log_10_ scale. A forward selection technique was performed by including the variable with *p*-values < 0.1. To test the adequacy of the multivariable regression model, a Hosmer-Lemeshow goodness of fit test was performed. Normally distributed data were presented as mean ± standard deviation while non-parametric data were presented as median and interquartile range (IQR). Data were analyzed using commercially available software (Prism 8, Graphpad, San Diego, CA and STATA v15.1, College Station, Texas).

## Results

### Study Population

Of the cats in the WF group, 16 were males and 13 were females. In the HCM group, 14 were males and 7 were females. In CC group, 8 were males and 3 were females. No significant differences in the proportion of males and females were found between either HCM or CC groups when compared to WF cats (*p* = 0.56, *p* = 0.47, respectively). Less WF cats were castrated or spayed (18/29) compared to the HCM (21/21, *p* = 0.0014) and CC groups (11/11, *p* = 0.02). Cats in the WF group also weighed less (4.0 kg, 3.7–4.5) than those in the HCM (5.8 kg ± 1.6, *p* < 0.001) and CC (5.5 kg ± 1.0, *p* = 0.0010) groups.

Table 1 summarizes the hematologic findings in all 3 groups of cats. Neutrophil count was significantly different among the 3 groups (*p* = 0.0012) with cats in the WF group having higher neutrophil counts compared to cats in the HCM group (*p* = 0.00060), There were no significant differences in platelet count or mean platelet volume among the 3 study groups (*p* = 0.92, *p* = 0.23, respectively).

**Table 1:** Summary of the complete blood count parameters of each study group.

**Study groups**	**WF (*N* = 29)**	**HCM (*N* = 21)**	**CC (*N* = 11)**	***P*** **-value**	
				**WF vs HCM**	**WF vs CC**
**Complete blood count parameters**					
White cell count × 10^9^/L (IQR or SD)	13.5 (±6.6)	7.48 (7.1–11.2)	8.8 (±2.5)	0.009	0.03
Neutrophil count × 10^9^/L (IQR or SD)	10.2 (±5.4)	4.0 (2.7–7.3)	5.8 (±2.6)	0.0006	0.12
Lymphocyte count × 10^9^/L (IQR or SD)	1.6 (0.9–2.8)	1.9 (1.2–2.9)	2.4 (±1.4)	0.47	0.25
Monocyte count × 10^9^/L (IQR or SD)	0.6 (±0.3)	0.3 (0.2–0.4)	0.3 (0.2–0.5)	0.0009	0.007
Hematocrit % (IQR or SD)	36.8 (±9.1)	43.5 (±4.8)	43.5 (±6.5)	0.67	0.03
Platelet count × 10^9^/L (IQR or SD)	204 (±87)	234 (±87)	208 (±106)	0.23	0.89
Mean platelet volume, fl (IQR or SD)	10.8 (10.3–11.5)	11.8 (10.6–12.5)	10.7 (±1.7)	0.08	0.48

*WF, wildfire cats; HCM, healthy cats with hypertrophic cardiomyopathy; CC, healthy cats without hypertrophic cardiomyopathy*.

### Patient Outcome

Of the 29 cats in the WF group, 25 (86.2%) survived to hospital discharge. Of the 4 cats that did not survive, all had a burn severity score of 3. One (3.4%) cat died of sudden death. Necropsy and histopathology of the heart showed LV concentric hypertrophy with evidence of lymphocytic and neutrophilic myocarditis. Lungs showed severe multifocal fibronecrotizing histiocytic pneumonia with intracellular pigment accumulation consistent with smoke inhalation. The remaining three (10.3%) cats were euthanized; one due to the development of acute kidney injury, another cat due to congestive heart failure (CHF) after receiving packed red blood cell transfusions and the third cat was euthanized due to respiratory distress unrelated to cardiac causes. Thoracic radiographs of this cat showed diffuse bronchointerstitial pattern without cardiomegaly. Table 2 details the therapies, sedatives, co-morbidites and outcomes of cats in the WF group. Anti-platelet drugs were administered following blood collection.

**Table 2:** Therapies, sedatives, co-morbidities and outcomes of 29 cats with naturally occurring wild fire injury and smoke inhalation.

**Severity of burn injuries**	**Number of cats**	**Antibiotics**	**Sedation**	**Analgesia**	**Antiplatelet drugs (started following platelet analysis)**	**Co-morbidity**	**Outcome**
Mild	7	Amoxicillin-clavulanic acid PO (1/7) Cefovecin sodium SC (3/7) None (4/7)	Dexmedetomidine (5/7) Alphaxalone (1/7) Ketamine (0/7) Midazolam (1/7)	Hydromorphone + Buprenorphine (3/7) Buprenorphine (4/7) Robenacoxib (2/7)	Clopidogrel (1/7) Aspirin (3/7) None (4/7)	Anemia (1/7)	Alive (7/7)
Moderate	7	Pradofloxacin (1/7) Ampicillin-sulbactam IV (1/7) Cefovecin sodium SC (4/7) Enrofloxacin IV (1 /7) None (3/7)	Dexmedetomidine (7/7) Alphaxalone (3/7) Ketamine (7/7) Midazolam (2/7)	Hydromorphone + Buprenorphine (6/7) Buprenorphine (1/7) Hydromorphone (1/7) Robenacoxib (1/7)	Clopidogrel (1/7) Aspirin (0/7) Clopidogrel + Aspirin (4/7) None (3/7)		Alive (7/7)
Severe^∧^	15	Amoxicillin-clavulanic acid PO (2/15) Pradofloxacin (1/15) Orbifloxacin (1/15) Ampicillin-sulbactam IV (1/15) Cefovecin sodium SC (7/15) Enrofloxacin IV (2/15) None (4/15) Unknown (2/15)	Dexmedetomidine (11/15) Alphaxalone (4/15) Ketamine (8/15) Midazolam (3/15) Unknown (3/15)	Hydromorphone + buprenorphine (9/15) Fentanyl Patch (1/15) Buprenorphine (1/15) Hydromorphone (1/15) Methadone (2/15) Robenacoxib (1/15) Unknown (3/15)	Clopidogrel (3/15) Aspirin (2/15) Clopidogrel + Aspirin (6/15) None (2/15) Unknown (2/15)	Anemia^*^ (2/15) Arrhythmia (1/15) Respiratory distress (2/15) Acute kidney injury (1/15)	Alive (11/15) Died (1/15) Euthanized (3/15)

* *Requiring blood transfusion*.

∧ *Two cats with no records of drugs administered*.

*PO, per os; IV, intravenous; SC, subcutaneous*.

### Echocardiographic Findings

Nine (9/29, 32.1%) cats in the WF group had evidence of MT. Expectedly, all HCM cats had significant MT in all 4 echocardiographic measurements compared to cats in the WF and CC groups (*p* < 0.01). No significant differences in these measurements were found between the WF and CC groups (Table 2). Of the 29 WF cats, 6 (20.7%) cats had evidence of LA enlargement compared to 6/21 (28.6%) cats in the HCM group (*p* = 0.74). None of the cats in the CC group had documented LA enlargement. LAuV was higher in WF group compared to CC group (*p* = 0.048) but was not different from that in HCM cats (*p* > 0.99). Only 1 WF cat had documented pericardial effusion. Spontaneous echocardiographic contrast with or without organized thrombus (SEC ± T) was found in 13/29 cats (44.8%) in the WF group. Of these 13 cats, 5/13 (38.46%) cats had an organized thrombus on echocardiography. None of the cats in the HCM group had documented SEC ± T. Echocardiographic parameters are summarized in Table 3.

**Table 3:** Patient characteristics of each study group.

**Study groups**	**WF (*N* = 29)**	**HCM (*N* = 21)**	**CC (*N* = 11)**	***p*** **-Value**		
				**WF vs HCM**	**WF vs CC**	**HCM vs CC**
**Echocardiographic data**						
2D IVSd, mm (IQR or SD)	4.8 (±1.0)	6.7 (±1.2)	4.8 (±0.5)	<0.0001	0.98	<0.0001
2D LVPWd, mm (IQR or SD)	5.2 (±0.8)	6.3 (±1.2)	4.9 (±0.5)	0.0009	0.71	0.0011
M mode IVS, mm (IQR or SD)	4.7 (±0.9)	5.6 (4.9–6.2)	4.4 (±0.6)	0.0097	>0.99	0.0071
M mode LVPW, mm (IQR or SD)	5.0 (±0.9)	5.8 (±0.8)	4.5 (±0.6)	0.0075	0.18	0.0004
Left atrial: aorta (IQR or SD)	1.4 (1.3–1.5)	1.5 (1.3–1.6)	1.4 (±0.1)	>0.99	>0.99	>0.99
Left auricular velocity, cm/s (IQR or SD)	59.2 (±14.7)	54.4 (48.2–61.0)	43.4 (39.3–52.5)	>0.99	0.048	0.14
Fractional shortening, % (IQR or SD)	51.5 (±9.7)	57.5 (±9.0)	57.2 (±14.4)	0.03	0.17	0.94
SEC ± T, number of cats	13/29 cats	0	0	NA		
	5/13 cats with thrombus				

*WF, wildfire cats; HCM, healthy cats with hypertrophic cardiomyopathy; CC, healthy cats without hypertrophic cardiomyopathy; IVSd, interventricular septal thickness at diastole; LVPWd, left ventricular posterior wall thickness at end-diastole; IVS, interventricular septum; LVPW, left ventricular posterior wall; SEC ± T, spontaneous echocardiographic contrast with or without organized thrombus in cardiac chambers*.

### Cats Exposed to Wildfires Had Evidence of Platelet Priming and Augmented Response to Adenosine Diphosphate

**Figure 2:**
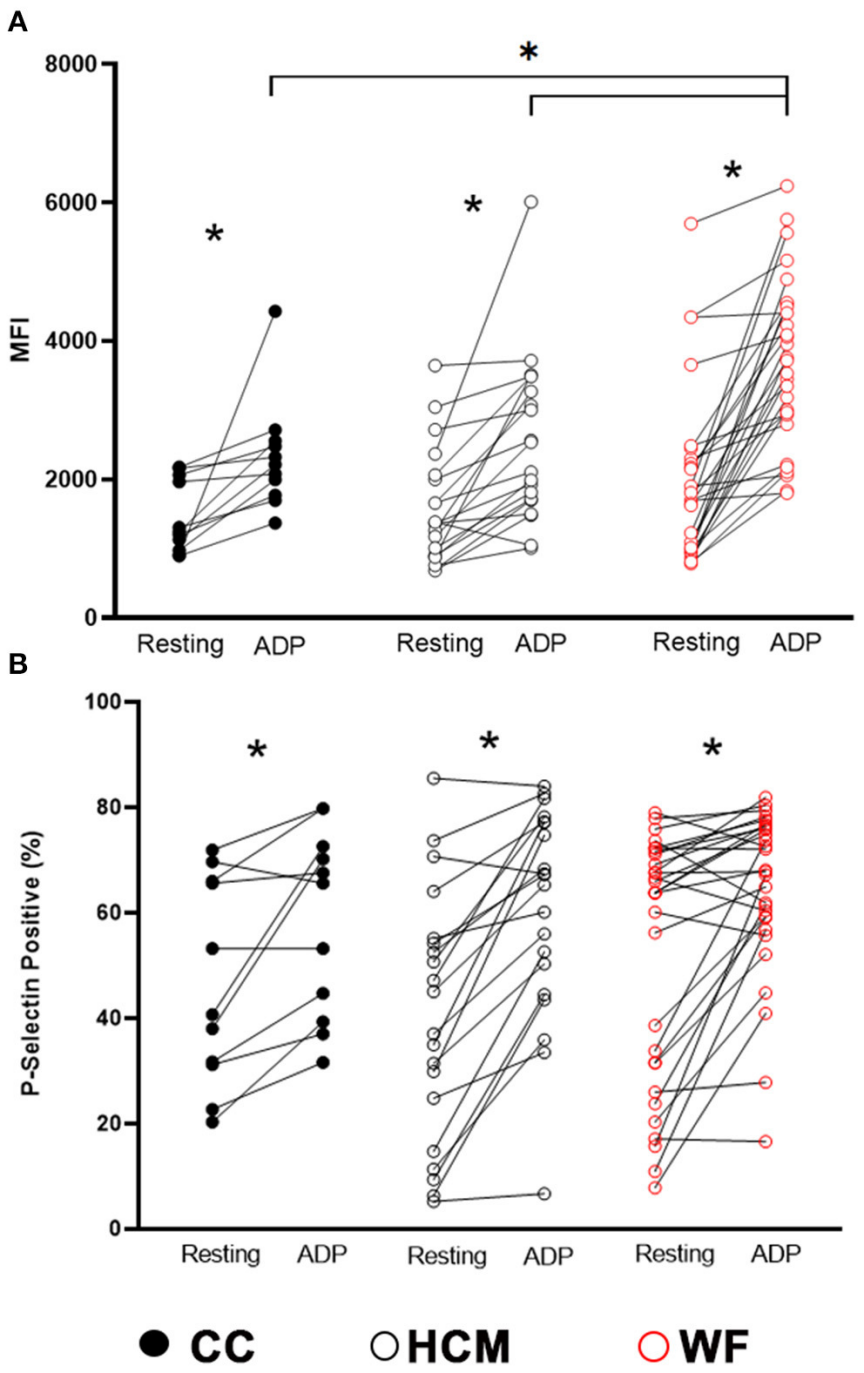
Increased platelet priming led to augmented response to adenosine diphosphate in cats with naturally occurring wildfire injuries and smoke exposure. Platelet activation, measured as P-selectin median fluorescence intensity (MFI) **(A)** and percentage of P-selectin positive events **(B)**, was evaluated by flow cytometry in 29 cats exposed to wildfires (WF), 21 cats with subclinical hypertrophic cardiomyopathy (HCM) and 11 control cats without HCM (CC). Platelet rich plasma was activated with 20 μM ADP and compared to unstimulated platelets (resting) within each group. ADP resulted in significant elevation in P-selectin expression in all 3 groups. WF cats had increased magnitude in P-selectin density (MFI) compared to cats in the HCM and CC groups in response to ADP. *p < 0.05.

ADP stimulation resulted in significant elevation in P-selectin (MFI and percent positive) when using paired *t*-test between resting and stimulated platelets (*p* < 0.05, Figure 2). Although we did not detect any differences in P-selectin expression in resting (unstimulated) platelets among the three study groups (*p* = 0.26; Figure 2), there was significantly higher ADP-induced P-selectin expression (MFI = 3,718; IQR: 2,866–4,517) in the WF group than those in HCM (MFI = 2,432; IQR: 1,707–3,168; *p* = 0.0002) and CC cats (MFI = 2,215; IQR 1,772–2,554, *p* = 0.0010; Figure 2A), suggesting that the response to ADP was more pronounced in the WF group. No significant difference was found between HCM and CC cats (*p* = 0.96).

Based on the linear mixed-effects model adjusted to the random effect of individual cats nested in the groups (CC vs. HCM vs. WF), ADP stimulation was significantly associated with elevated P-selectin MFI (coefficient = 871.8, *p* = 0.007) and positive events (%) (coefficient = 11.9, *p* = 0.034). When factoring in the interactions between groups (CC vs. HCM vs. WF) and ADP stimulation, ADP stimulation was significantly associated with elevated P-selectin MFI in WF cats (coefficient = 895.8, *p* = 0.018), suggesting platelet priming in the WF group. The *post-hoc* pairwise comparisons noted the significant differences in P-selectin MFI between cats in the WF and CC groups (*p* = 0.015), and WF and HCM groups (*p* = 0.016). No significant association of P-selectin positive events (%) was noted between the group of cats.

**Figure 3:**
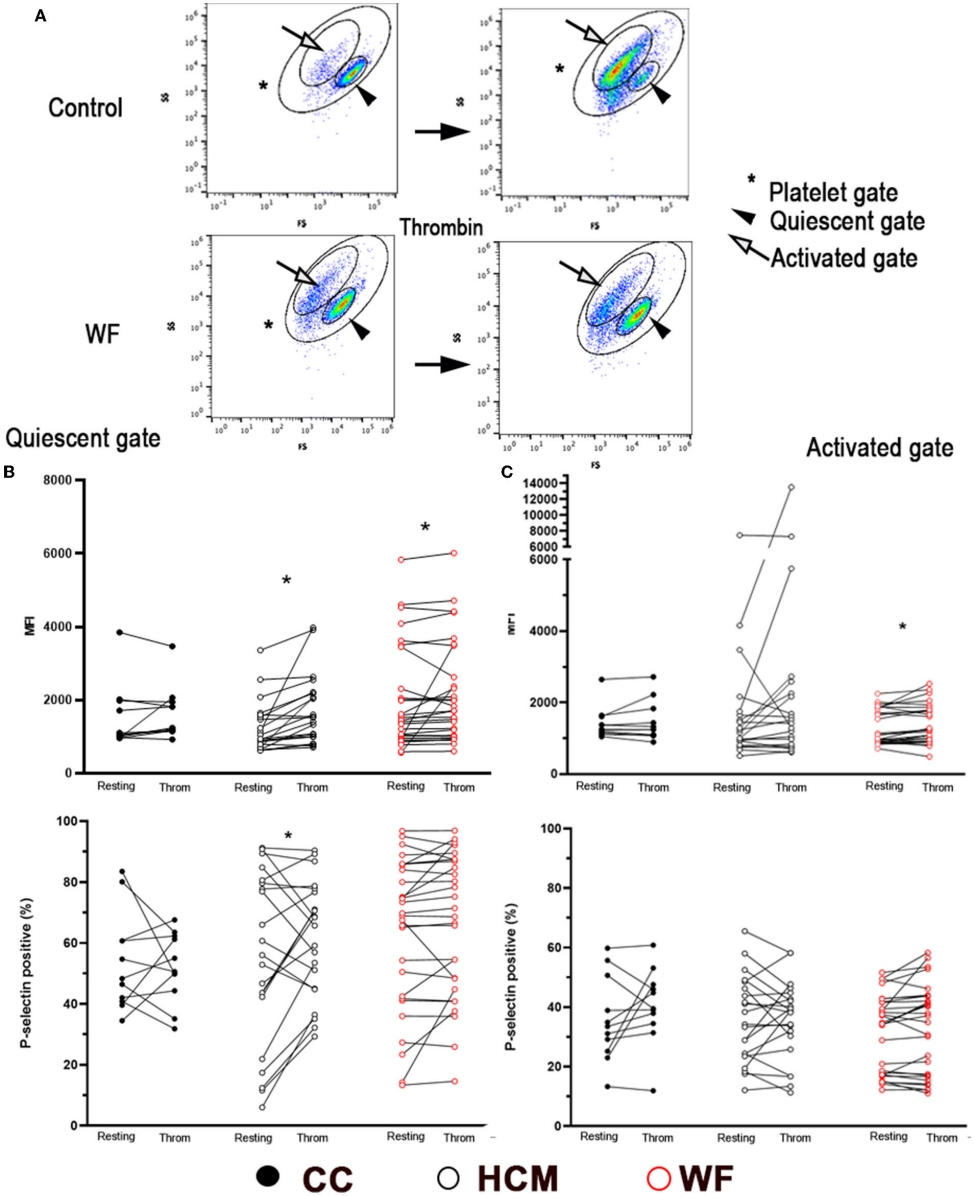
Representative scatter dot plot diagrams, gating strategy and P-selectin expression in thrombin-activated platelets. **(A)** Platelets were first identified by forward (FS) and side scatter (SS) properties (*Platelet gate). With thrombin activation (0.01 U/ml), events were further gated based on scatter properties to either Quiescent (arrowhead) or Activated (arrow). Note the shift of cells from the Quiescent to Activated gates following thrombin treatment in a control cat while the number of events was similar in either gate in a cat exposed to wildfires. Platelet activation, measured as percentage of P-selectin positive events, or median fluorescence intensity (MFI) was compared before and after thrombin stimulation in 29 cats exposed to wildfires (WF), 21 cats with subclinical hypertrophic cardiomyopathy (HCM) and 11 control cats without HCM (CC). **(B)** In the Quiescent gate, thrombin resulted in elevated P-selectin in HCM and WF groups but no difference was noted among the three groups. **(C)** Only cats in the WF group had elevated levels of P-selectin (MFI) in the Activated gate following thrombin stimulation. *p > 0.05.

Thrombin-stimulated platelets were further gated, according to changes in forward- and side-scatter properties, to Quiescent (Q) and Activated (A) gates as shown in Figure 3A. The paired *t*-test or Wilcoxon tests revealed that thrombin stimulation resulted in a significant shift of events from Q to A gates (Figure 3A). This was most notable in cats in the CC group (*p* < 0.0001) and HCM group (*p* = 0.0028; Supplementary Figure 1A). Interestingly, cats in the WF group did not have a similar shift of events from the Q to A gates (*p* = 0.070). When comparing P-selectin MFI between resting and thrombin-activated platelets within the Q gate, only HCM (938; IQR 750–1,564 vs. 1,522; IQR 996.5 vs. 2,197, *p* < 0.0001) and WF groups (1,441; IQR 949.0–2,879 vs. 1,707; IQR 1,014–3,056, *p* = 0.030) had augmented response to thrombin but not the CC group (1,052; IQR 973–1,981 vs. 1,242; IQR 1,161–1,941, *p* = 0.32). Only cats in the HCM group had elevated P-selectin-positive platelets within the Q gate (Resting = 56% ± 28.43 vs. Thrombin = 61.30% ± 19.10, *p* = 0.0305; Figure 3B).

When comparing platelets within the activated gate, only platelets from cats in the WF group had significant upregulation in response to thrombin (Figure 3C; P-selectin MFI 993; IQR: 872–1,770 vs. 1,188; IQR: 911.5–1,793, *p* = 0.025) while no differences in P-selectin expression (% positive) were found among the 3 groups with thrombin stimulation (Figure 3C).

The linear mixed-effects model was also constructed with thrombin stimulation of platelets in the Q and A gates, adjusted to the random effect of individual cats nested in their groups. In this model, thrombin stimulation, group of cats (CC vs. HCM vs. WF), and their interaction were not significantly associated with changes in P-selectin MFI and positive event % in both Q and A gates.

### Cats With Spontaneous Echocardiographic Contrast and/or Intracardiac Thrombosis Had Increased Platelet Activation and Platelet Priming

**Figure 4:**
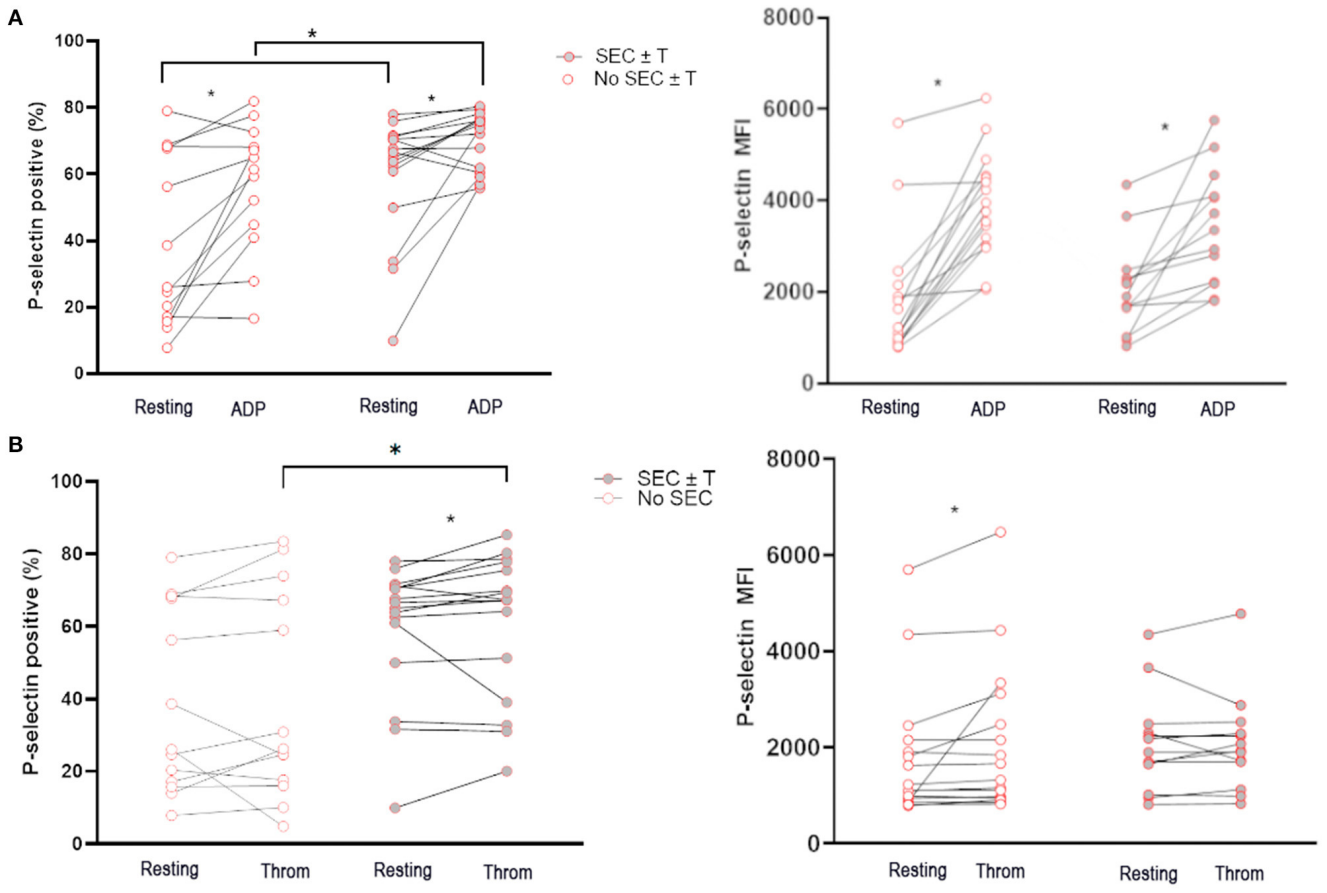
Cats with naturally occurring wildfire related (WF) injuries had increased activated platelets and platelet priming. Platelet activation in 29 WF cats with or without spontaneous echocardiographic contrast with or without intracardiac thrombi (SEC ± T) was measured as either percentage of P-selectin-positive platelets or P-selectin median florescence intensity (MFI) at rest, or in the presence of 20 μM ADP **(A)** or 0.01 U/ml thrombin **(B)**. **(A)** Cats with SEC ± T had increased amount of P-selectin positive platelets in unstimulated (resting) platelets. **(A,B)** There was also evidence of increased platelet priming in cats with SEC ± T given that higher percentages of P-selectin positive platelets were found in cats with SEC ± T compared to those without following treatment of platelets with ADP or thrombin. *p < 0.05.

Unstimulated platelets from cats in the WF group with documented SEC ± T had a higher percentage of P-selectin-positive platelets than those without SEC ± T (65.8%; IQR 52.65–70.95 vs. 26.2%; IQR 16.80–70.55, *p* = 0.049; Figure 4A). While platelets from both groups responded to ADP, the magnitude of P-selectin elevation was higher in cats with SEC ± T compared to those without SEC ± T (70.33% ± 8.64 vs. 56.55% ± 19.40, *p* = 0.030), as measured by % positive platelets. Interestingly, platelets from WF cats with SEC ± T were also noted to be more responsive to thrombin (Q gate: 65.8%; IQR 52.65–70.95 vs. 67.25%; IQR 42.05–77.13, *p* = 0.045) and thrombin-stimulated platelets had higher percentage P-selectin than those in cats without SEC ± T (60.98% ± 19.97 vs. 39.93% ± 28.57, *p* = 0.036; Figure 4B). This was, however, not reflected, in P-selectin MFI.

### Cats With Spontaneous Echocardiographic Contract and/or Intracardiac Thrombosis Had Persistent Platelet Activation in Response to Arachidonic Acid (AA)

**Figure 5:**
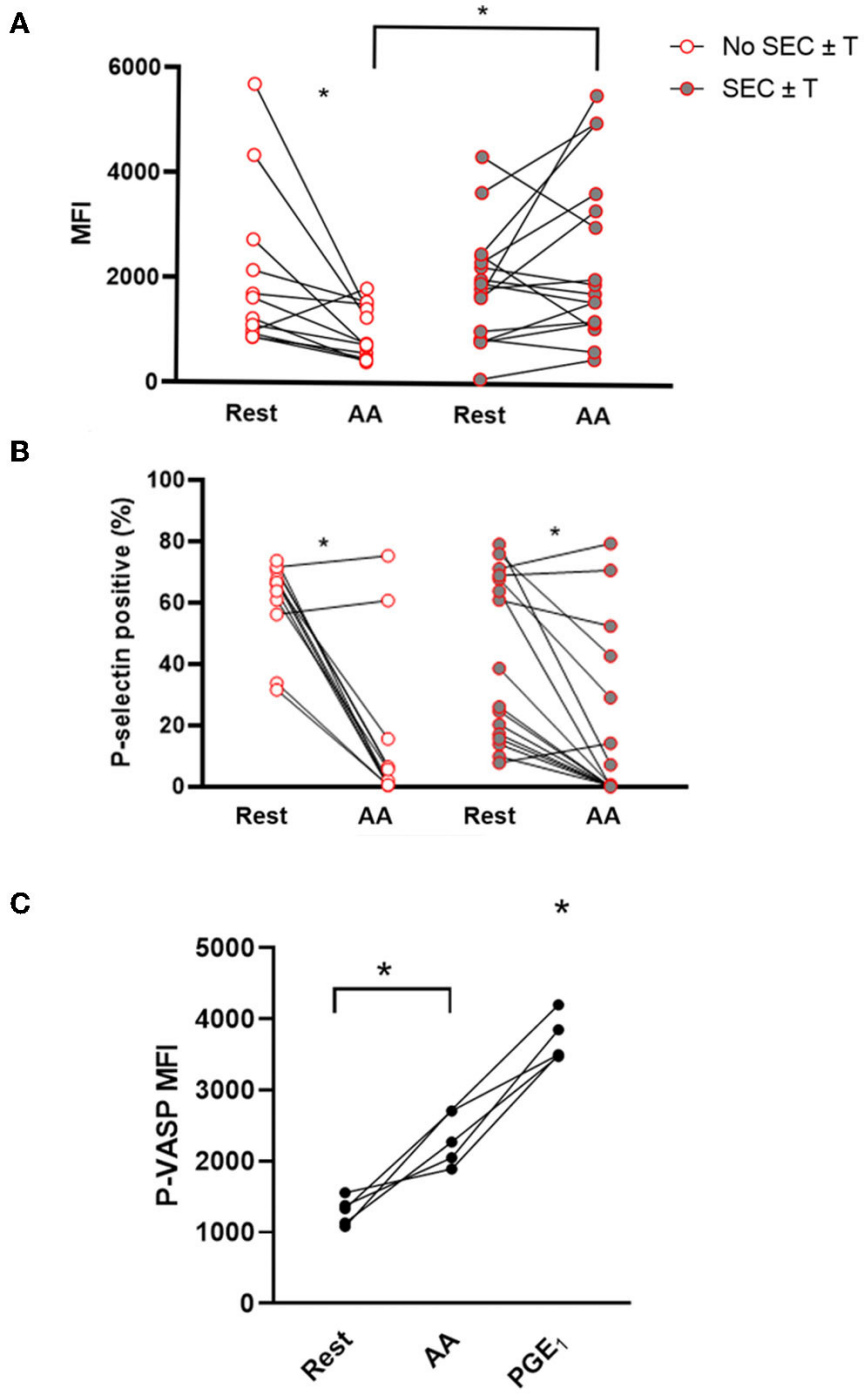
*Ex vivo* treatment of platelets with arachidonic acid results in platelet inhibition but not in those with spontaneous echocardiographic contrast with or without intracardiac thrombi. Platelet activation was assessed in 29 cats with wildfire (WF) injuries, measured as P-selectin median fluorescence intensity (MFI) **(A)** or percentage (%) of P-selectin positive platelets **(B)** at rest and in the presence of 1 μM arachidonic acid (AA) by flow cytometry. While AA caused a significant decrease in percentage of P-selectin positive platelets in cats with or without SEC ± T **(A)**, those with SEC ± T sustained P-selectin density (MFI) following AA treatment compared to those without SEC ± T, which had significantly lower MFI **(B)**. **(C)** Platelets from five healthy cats were unstimulated (rest) or treated with either 1 μM AA or 10 μM PGE_1_. Phosphorylation of intracellular vasodilator-stimulated phosphoprotein (P-VASP) was measured by flow cytometry. Elevation in P-VASP MFI in the presence of AA indicates significant platelet inhibition. *p < 0.05.

*Ex vivo* platelet activation in response to AA was further characterized in the WF group. Unexpectedly, AA led to a significant decrease in P-selectin MFI compared to unstimulated (resting) platelets in those without SEC ± T (Resting = 2,020.0 ± 1,538 vs. AA = 973.5 ± 504.0, *p* = 0.026). This, however, was not the case in cats with SEC ± T (Resting = 1,696; IQR 970.50–2,265 vs. AA = 1,341; IQR 620.81–1,841 *p* = 0.30; Figure 5A). Cats with SEC ± T had persistent elevation in P-selectin density, as measured by MFI, compared to those without SEC ± T (*p* = 0.0059; Figure 5A). AA, however, consistently decreased the number of P-selectin positive platelets in cats with or without SEC ± T (SEC ± T; Resting = 41.32% ± 26.98 vs. AA = 18.57 ± 27.56%; No SEC ± T; Resting = 61.08% ± 14.11 vs. 14.58% ± 25.53, *p* = 0.0004. *p* = 0.0024, respectively; Figure 5B). To further confirm the inhibitory effects of AA, flow cytometric analysis of P-VASP within platelets from 5 healthy cats were analyzed in the presence or absence of 1 μM AA or prostaglandin E1 (PGE_1_) as positive control. We found that treatment of platelets with 1 μM AA for 15 min resulted in significant increase in P-VASP (MFI = 2,327 ± 378.0) compared to unstimulated platelets (P-VASP MFI = 1,296 ± 193.0; *p* = 0.012; Figure 5C).

### Cats Exposed to Wildfires Had Elevated Circulating Platelet-Derived Microvesicles Compared to Healthy Controls

**Figure 6:**
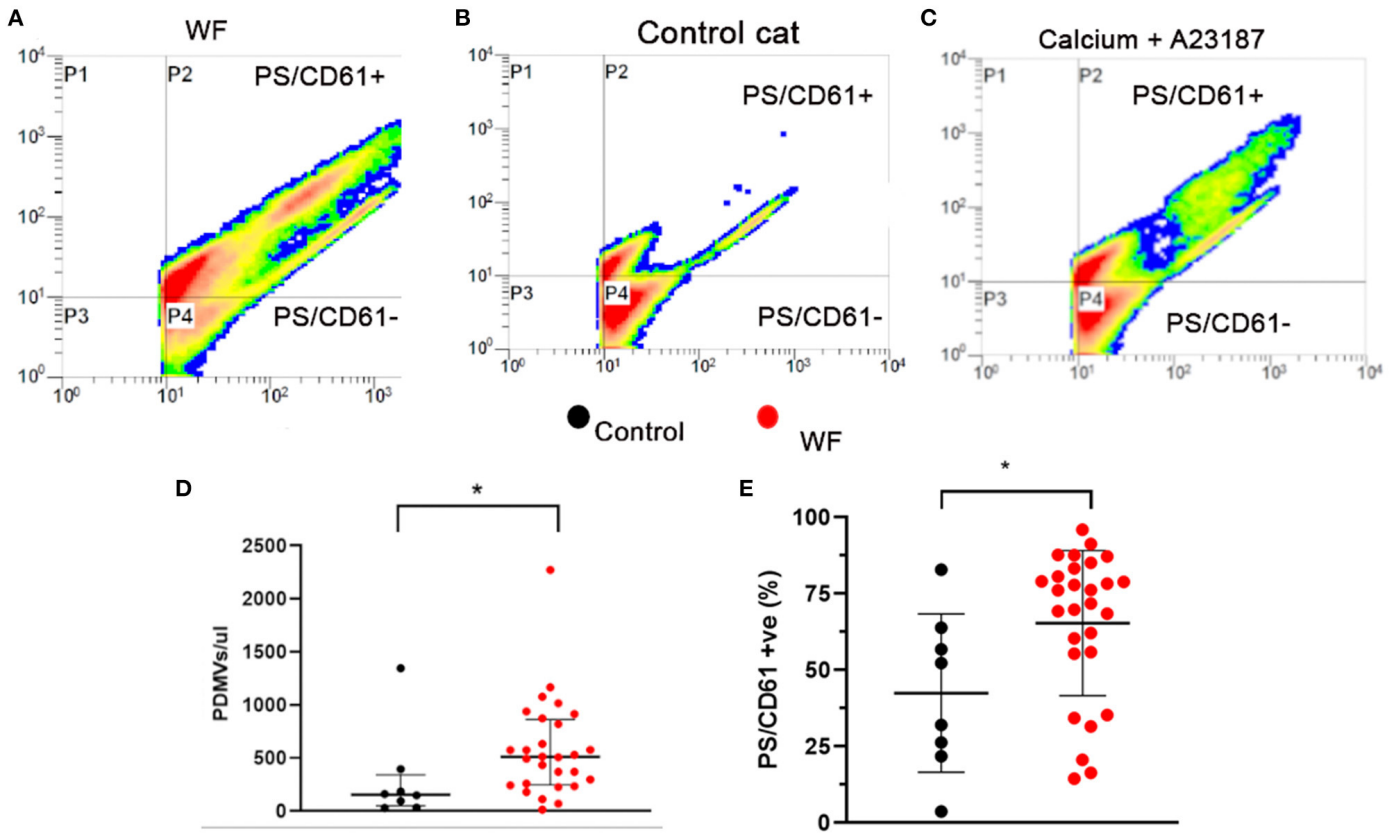
Cats with naturally occurring wildfire injuries (WF) had elevated circulating platelet-derived microvesicles (PDMV). PDMV was measured in platelet poor plasma in 29 WF cats and 8 healthy control cats by flow cytometry. **(A–C)** Representative scatter dot plot diagrams of flow cytometric analysis of PDMV identified based on size, side scatter properties and co-expression of integrin β3 (CD61) and phosphatidylserine (PS) – P2. **(A)** Representative scatter plot generated from a cat with wildfire-related injuries, **(B)** from a control cat without hypertrophic cardiomyopathy and **(C)** positive control generated by activating platelets with 2 mM calcium and the calcium ionophore, A23187 (2.5 μM), which showed a significant increase in PDMV compared to resting sample. **(D,E)** WF cats not only had increased number of PDMV but a greater percentage of them had externalized PS compared to healthy controls. *p < 0.05.

Representative gating of PDMV identified by size, scatter properties, as well as CD61, and annexin V are demonstrated in Figures 6A–C. Scatter profile and PDMV numbers from platelets treated with calcium and A23817 were similar to those seen in cats from the WF group (Figures 6A,C). We found that WF cats had significantly elevated concentrations of circulating PDMV compared to CC cats (510.7/μl; IQR 246.5–860.9 vs. 155.2/μl; IQR 47.95–341.6, *p* = 0.021; Figure 6D). A higher percentage of PDMV with PS on the outer leaflet (% positive) was found in WF cats compared to CC cats (65.39% ± 23.78 vs. 42.46% ± 25.90, *p* = 0.024; Figure 6E). Neither the concentration of PDMV (514.5/μl; IQR 333.5–925.2 vs. 507.0/μl; IQR 179.6–631.8, *p* = 0.34) nor the percentage of PS-positive PDMV (63.23% ± 25.62 vs. 67.25% ± 22.79, *p* = 0.50) were found to be different among WF cats with and without SEC ± T.

### Echocardiographic Changes Had no Impact on Platelet Priming, Spontaneous Echo Contrast or Intracardiac Thrombosis in Cats Exposed to Wildfires

**Figure 7:**
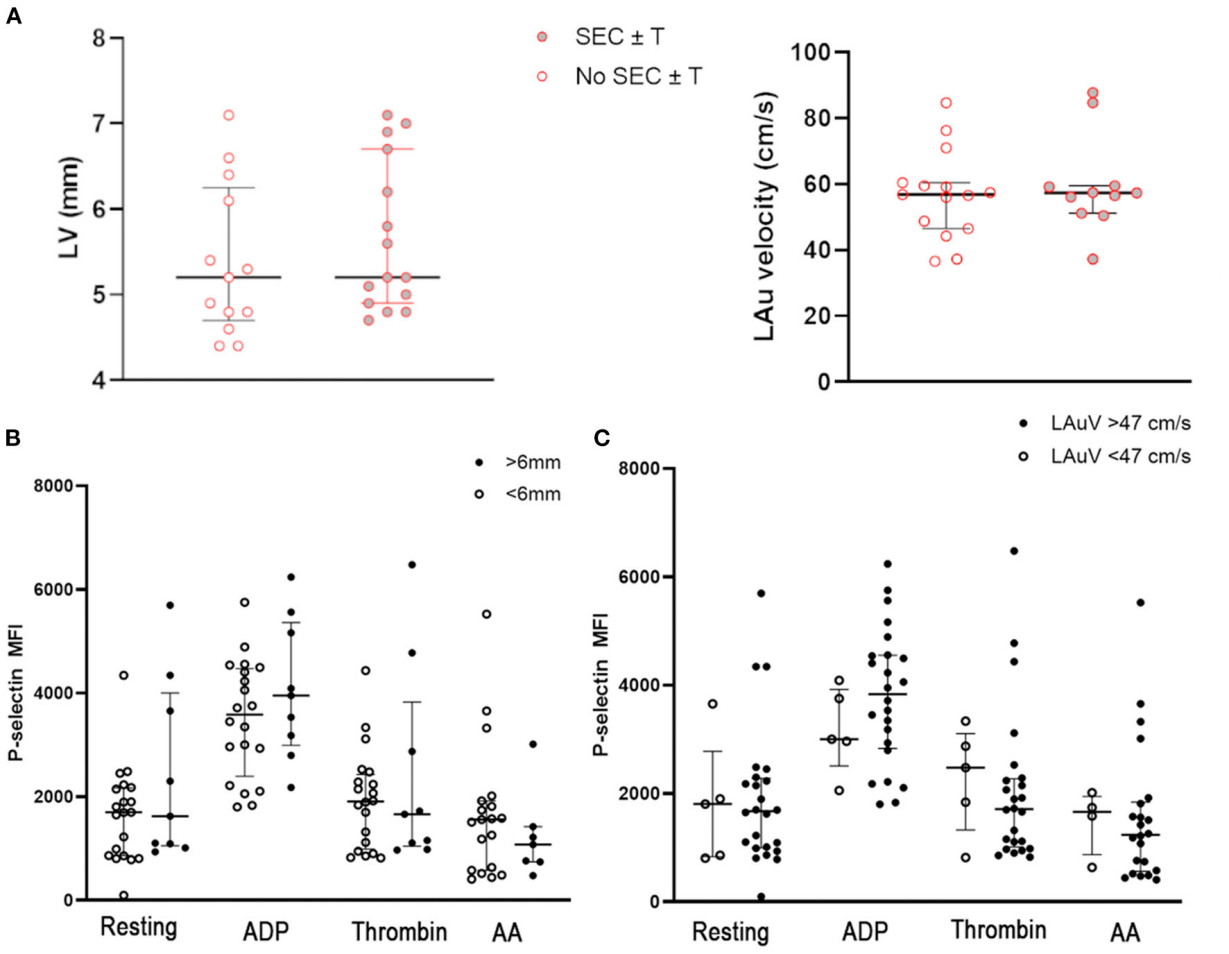
**(A)** Myocardial thickness, measured based on maximum wall thickness of the left ventricle (LV) and left atrial function, assessed by left auricular flow velocity (LAuV) did not differ among cats exposed to wildfires with or without spontaneous echocardiographic contrast ± intracardiac thrombi (SEC ± T). **(B)** Platelet activation in 29 cats, measured as P-selectin median florescence intensity (MFI) at rest or in the presence of 20 μM ADP, thrombin or 1 μM arachidonic acid (AA). Platelet activation did not differ among cats with evidence of myocardial thickness. **(C)** Similarly, there was no evidence of increased platelet priming in cats with normal or low left auricular flow velocity (LAuV).

Since cats with subclinical HCM have previously been reported to have increased platelet activation and increased platelet priming, we sought to assess the effects of myocardial changes on platelet activation and the formation SEC ± T within the WF group (19, 20). Cats with SEC ± T did not have significant differences in LV wall thickness (5.36 mm ± 0.89 vs. 5.67 mm ± 0.88, *p* = 0.41) or LAuV (59.79 cm/s ± 14.51 vs. 58.66 cm/s ±15.31, *p* = 0.85) compared to those without SEC ± T (Figure 7A). We also did not find significant differences in platelet activation and response to platelet agonists in those with documented MT. P-selectin in resting platelets were not significant different between cats with and without MT (MFI: 1,623; IQR 1,053–4,000 vs. MFI: 1,699; IQR 862.8–2,171, *p* = 0.35). Similarly, response to ADP (MFI: 4,077 ± 1,342 vs. MFI: 3,506 ± 1,128, *p* = 0.29), thrombin (MFI: 2,414 ± 1,961 vs. MFI: 1,939 ± 954.8, *p* = 0.51) and AA (MFI: 1,077; IQR 742–1,422 vs. MFI: 1,558; IQR 578–1,922, *p* = 0.39) were comparable between the 2 groups (Figure 7B). There were also no differences in P-selectin expression among WF cats with normal LAuV (>47 cm/s) compared to those with low LAuV (<47 cm/s) in resting (MFI: 1,673; IQR 996–2,282 vs. MFI: 1,808; IQR 835–2,779, *p* = 0.81), ADP- (MFI: 3,835; IQR 2,832–4,551 vs. MFI: 3,004; IQR 2,510–3,922, *p* = 0.32) thrombin- (MFI: 1,713; IQR 1,014–2,273 vs. MFI: 2,477; IQR 1,329–3,196, *p* = 0.48) or AA- (MFI: 1,238; IQR 562.8–1,841 vs. MFI: 1,664; IQR 872.5–1,947, *p* = 0.43) activated platelets (Figure 7C). Similar findings were noted in P-selectin positive platelets.

### Platelet Activation in Response to Arachidonic Acid (AA) Is Associated With Spontaneous Echocontrast and Intracardiac Thrombosis in Cats With Naturally Occurring Wildfire Injuries

To determine the association between platelet activation, severity of burn injuries and intracardiac thrombosis (SEC ± T), univariate and multiple logistic regression analyses were performed. We found a significant association between SEC ± T and platelet activation in response to AA (*p* = 0.0066). However, no association between SEC ± T and severity of burn injury (*p* = 0.21) was found. Other variables such as resting P-selectin expression, response to ADP, measured as log_10_ MFI fold change, and neutrophil count were not associated with SEC ± T (*p* > 0.5). Simple logistic regression analysis revealed that the magnitude of platelet response to AA (log_10_ MFI fold change) predicted clot formation (*R*^2^ = 0.24, OR = 48.3, 95% CI 1.31–1,779.2, *p* = 0.0022). Further analysis by multiple logistic regression to predict SEC ± T based on severity of burn injuries, resting P-selectin expression (MFI), magnitude of platelet response to AA or ADP (log_10_ MFI fold change), and neutrophil count was performed. Platelet response to AA remained to predict formation of SEC ± T (OR = 42.5, 95% CI 1.2–1,496.5, *p* = 0.039). Simple and multiple logistic regression analysis revealed none of the variables predicted survival in cats in the WF group.

## Discussion

Our findings indicate that naturally occurring wildfire related thermal burn injuries and smoke exposure resulted in the priming of platelets that potentiated platelet activation and shedding of PDMV in cats. Persistent platelet activation in response to AA was also independently associated with intracardiac thrombus in this population of cats.

The findings of this study open a dialogue about the impact of natural wildfires and air pollution on the well-being of humans, wildlife, and domestic animals. There is mounting epidemiologic data relating poor air quality from wildfires to growing incidences of human cardiovascular diseases, CHF, out-of-hospital cardiac arrest and cerebrovascular events (2, 3, 5, 25–30). While robust data exists in both animal models and human studies documenting the procoagulant effects of fine particulate matter (PM_2.5_) exposure, the role of platelets in propagating a hypercoagulable state and subsequent thrombosis in a natural setting has never been established. Our data show that wildfire-related injuries and smoke inhalation results in the priming of platelets. Priming occurs when an initial stimulus, known as a primer, counteracts the intrinsic regulatory mechanisms to potentiate the activation of platelets upon subsequent exposure to physiologic agonists. This causes unrestrained platelet activation, aggregation, and sustained thrombus formation (31). Similar findings have also been shown in experimental models of PM_2.5_ exposure. Studies demonstrated that mice exposed to concentrated ambient particulate matter not only have elevated inflammatory cytokines and PDMV, but also have platelets with increased fibrinogen binding and sensitivity to thrombin, both of which are indicative of platelet priming (17, 32). While it is difficult to ascertain the extent of PM_2.5_ inhalation in our population of cats, forecast models in Northern California's Butte County and the surrounding regions during the time of enrollment estimated toxic levels of PM_2.5_ (33). In addition, cats rescued from the California Camp Fire originated from an urbanized environment and likely inhaled smoke comprised of noxious gases and potent PM_2.5_.

Although our study did not assess the presence of platelet primers in cats with wildfire related injuries, increased exposure to specific platelet primers in the wildfire cats could explain the augmented platelet response seen in the WF group. Human burn victims have increased levels of endogenous platelet priming molecules like thrombopoietin, epinephrine, and vascular endothelial growth factors (34–36). Other less well described primers such as dioxins, benzimidazoles and lipopolysaccharide (LPS) are known PM_2.5_-bound trace elements. Dioxins have previously been shown to prime human platelets (37). PM_2.5_-bound LPS can also modulate splenocyte immune response and exacerbate airway inflammation by TLR4 and−2 pathways (38–40). We previously showed that *ex vivo* exposure of canine platelets to LPS primes platelets to respond to ADP via platelet TLR4 to potentiate the cyclooxygenase pathway leading to excessive release of thromboxane A_2_ (TxA2) (23). We suspect that platelet TLR4 may play a role in platelet priming in wildfire-related injuries but further studies are needed.

Arachidonic acid was chosen as an agonist to assess platelet activation in cats in the WF group to determine if aspirin, a COX-1 inhibitor, would be indicated in those with intracardiac thrombosis. While exogenous addition of AA generally resulted in platelet inhibition in our study, persistent platelet P-selectin expression and density in response to AA was found to be independently associated with intracardiac thrombosis. Two human studies of flame-related or electrical burn events found a similar response to AA stimulation; the mechanism for this response, however, has not been elucidated (7, 41). We found that similar treatment of AA resulted in elevation in P-VASP, which indicates that prolonged exposure of high-dose AA in feline platelets inhibited platelets. This could be due to production of high concentrations of PGE_1_ and PGE_2_ in the platelet milieu and subsequently increasing cyclic adenosine monophosphate cAMP production and phosphorylation of VASP inhibiting platelet function (24, 42). AA, a phospholipid released by the soluble enzyme phospholipase A_2_, is catalyzed by cyclooxygenase (COX) to produce TxA_2_, prostacyclin (PGI_2_) and prostaglandin E2 (PGE_2_). While TxA_2_ enhances platelet activation via TPα receptors, PGI_2_ and PGE_2_ inhibit platelets by binding to their respective EP2 and EP4 receptors, leading to increased intracellular cAMP levels (14, 43). There are two plausible explanations to this observed biphasic response to AA. First, this concentration of AA was chosen based on a previous study, which evaluated whole blood platelet aggregometry in healthy cats (44). Interestingly, that study did not show any modulation of aggregation after a total analysis time of 6 min, further supporting that prolonged treatment to AA inhibits feline platelets. Second, it is conceivable that circulating platelets may encounter platelet primers released or shed from the endothelium or established thrombi as they pass through the LA. Platelet-derived primers released from activated platelets may also further potentiate platelet response. For example, low concentrations of PGE_2_ released from activated platelets have been shown to potentiate platelet aggregation by priming phosphokinase C and inhibiting adenylcyclase (45, 46). This may shift the inhibitory pathway of AA to a stimulatory one that favors TxA_2_ production. Given the biphasic response to AA noted in our study, further studies are needed to delineate the mechanisms of AA-mediated platelet activation noted in cats with intracardiac thrombosis and to investigate the efficacy of anti-platelet therapies in preventing thrombosis in cats with wildfire related injuries.

In agreement with previous studies, we found that cats with subclinical HCM had evidence of increased platelet priming compared to healthy controls. We chose cats with subclinical HCM as a control group since previous studies had found that subclinical HCM, LV cavity obliteration and decreased diastolic function were associated with increased platelet priming, platelet activation and PDMV shedding (18–20, 47). In addition, compromised LA function secondary to HCM, which leads to blood flow stasis, has also been proposed to facilitate the formation of SEC ± T and cardiogenic thromboembolism (22). Interestingly, the presence of MT was not associated with platelet activation while SEC ± T also occurred in the absence of LA dysfunction among WF cats. In addition, cats in the WF group had platelets with more exaggerated response to ADP compared to those in HCM group. These findings suggest that increased platelet priming and SEC ± T in WF cats were unlikely to be related to their transient cardiomyopathies. The myocardial changes noted in WF cats could be due to myocardial edema, myocarditis from systemic inflammation or catecholamine surges following wildfire injuries (1). Increased platelet priming and activation could, therefore, occur in parallel with the observed transient cardiomyopathies as a result of systemic inflammation due to smoke inhalation and thermal burn injuries. In contrast, HCM, which causes primary myocardial structural changes, augments the components of Virchow's triad. While cats in the WF group may have similar echocardiographic indications like LA dilation or presence of SEC ± T to initiate anti-platelet therapy, it is important to note the differences in the underlying causes of thrombosis between HCM and wildfire injuries (48, 49). For that reason, aspirin, which was shown to be inferior to clopidogrel in preventing recurrent thrombosis in HCM cats, may be a more appropriate antiplatelet therapy than clopidogrel in cats with wildfire-related injuries.

While our study did not evaluate specific inflammatory biomarkers, increased neutrophil counts found in the WF group is indicative of a proinflammatory state. Increases in interleukin-10 and macrophage colony-stimulating factor, alongside increases in circulating PDMV have been in found in mice exposed to particulate matter and human burn victims (17, 41). Similarly, cats in the WF group had substantial elevation in circulating PDMV, which may further promote thrombosis by facilitating the assembly of thrombin generating complexes on externalized PS (14, 15, 50). P-selectin, found on the membrane of platelet α-granules, is expressed on the plasma membrane upon platelet activation and degranulation. In mice, upregulation of platelet P-selectin promotes platelet-leukocyte interaction by binding to P-Selectin Ligand-1 on leukocytes, thus triggering the upregulation of tissue factor on leukocytes and initiating the extrinsic coagulation cascade in the absence of vascular injury (15, 51, 52). In 1 study that performed intratracheal instillation of carbon nanotubes as a surrogate for air pollution, P-selectin neutralization abrogated platelet-leukocyte aggregations and procoagulant microvascular tissue factor activity in mice (53). This highlights the role that platelet P-selectin may play in linking hemostasis and inflammation following wildfire injuries. However, the molecular mechanisms involved in platelet-leukocyte interaction are highly species dependent and further investigations are needed to further elucidate the role of platelet P-selectin in thrombosis and inflammation in cats.

Platelets from all 3 groups of cats had increased activation at resting state, which could dampen their response to agonists. This likely explains why cats in the CC and WF groups had similar numbers of P-selectin-positive platelets in the presence or absence of thrombin. This increased activation may be attributed to patient stress and epinephrine release despite our efforts to minimize stress by gentle restraint and administration of sedation when necessary. The level of P-selectin expression observed in resting platelets in our HCM healthy controls is consistent with our previous findings hence it is considered an appropriate baseline in our study (19, 20). Furthermore, given the observed response following ADP agonism across all 3 groups, the pre-activation of platelets at rest did not appear to impact this study. The lack of elevation in P-selectin in response to thrombin despite the characteristic changes in forward- and side-scatter profile on flow cytometry suggests that formation of large aggregates, especially in the control group, could falsely decrease the number of P-selectin positive platelets and overestimate density of P-selectin, as measured by MFI. Hence this data should be interpreted with caution.

This study has several limitations. First, we did not investigate the mechanisms of platelet activation due to wildfire-related injuries and smoke exposure. We also did not evaluate the secondary and tertiary hemostasis so the impact of wildfire-related injuries on global coagulation cannot be confirmed. Second, pro-inflammatory mediators and biomarkers for assessment of smoke exposures like carbon monoxide, aromatic hydrocarbons and particulate matter levels, or plasma markers of platelet activation, were not measured given the limited volume of blood that we could safely collect from the study population. Also, due to the emergency presentation of patients, the duration and extent of injuries and smoke inhalation in these cats could not be accurately determined. However, attempts were made to standardize the time of sample collection following presentation. In addition, the lack of a validated system to estimate the extent of burn severity in cats might have greatly limited our ability to identify associations between thrombosis and burn severity. Our assessment was largely based on review of medical records and subjective assessment of photographic documentation hence total body surface area affected could not be estimated in our cohort of animals. In human patients, estimated burn injuries affecting >6% of total body surface area were shown to be associated with systemic hypercoagulability and early coagulopathy (54). Furthermore, various therapies such as antimicrobials and co-morbidities in the WF group may contribute to changes in platelet function although logistic regression analyses did not find significant associations between burn severities, leukocyte count and platelet activation. Also, given that cats with HCM were shown to have hyper-reactive platelets, they were chosen as a control group to demonstrate that platelet priming as a result of wildfire smoke exposure and thermal burn injuries occurred independently of underlying cardiomyopathies. However, since clinical or echocardiographic characteristics at the time of enrollment could not differentiate cats in the WF group with transient MT from HCM, additional factors such as co-morbidities or underlying heart disease also may contribute to our findings (1). Lastly, *in vitro* platelet activation and *ex vivo* formation of platelet aggregates during the analysis process is a limitation.

In conclusion, persistent platelet activation, especially in response to ADP, and shedding of PDMV is present following naturally occurring wildfire exposure and thermal burn injuries in a population of domestic cats. This may exacerbate systemic inflammation and hemostatic derangement resulting in cardiovascular events and thrombosis, which is associated with persistent platelet response to AA. Aspirin may be the preferred anti-platelet therapy of choice for a subset of feline patients with confirmed thrombosis but further studies are required to delineate additional mechanisms between inflammation and thrombosis, especially in regard to the COX-1 pathway.

## Data Availability Statement

The raw data supporting the conclusions of this article will be made available by the authors, without undue reservation.

## Ethics Statement

The animal study was reviewed and approved by Institutional Animal Care and Use Committee. Written informed consent was obtained from the owners for the participation of their animals in this study.

## Author Contributions

RL and AT secured funding for this study, participated in study design, gathering data, data analysis, and drafting of manuscript. JS, SE, CG-H, and AS took part in sample collection, gathering data, and study design. YU, AT, NN, MH, and SH contributed to collection of samples, analysis of samples, and data analysis. All authors participated in editing of the manuscript.

## Funding

This study was funded by the Center for Companion Animal Health (2019-30-F) of the University of California, Davis. Open access publication of this manuscript was partially funded by the UC Davis Library Open Access Publication Funds.

## Conflict of Interest

The authors declare that the research was conducted in the absence of any commercial or financial relationships that could be construed as a potential conflict of interest.

## Publisher's Note

All claims expressed in this article are solely those of the authors and do not necessarily represent those of their affiliated organizations, or those of the publisher, the editors and the reviewers. Any product that may be evaluated in this article, or claim that may be made by its manufacturer, is not guaranteed or endorsed by the publisher.
