# Supplementary figures and images for: Platelet Priming and Activation in Naturally Occurring Thermal Burn Injuries and Wildfire Smoke Exposure Is Associated With Intracardiac Thrombosis and Spontaneous Echocardiographic Contrast in Feline Survivors
Source: Front Vet Sci. 2022 Jul 14;9:892377. doi: 10.3389/fvets.2022.892377 (PMC9329816; doi:10.3389/fvets.2022.892377)

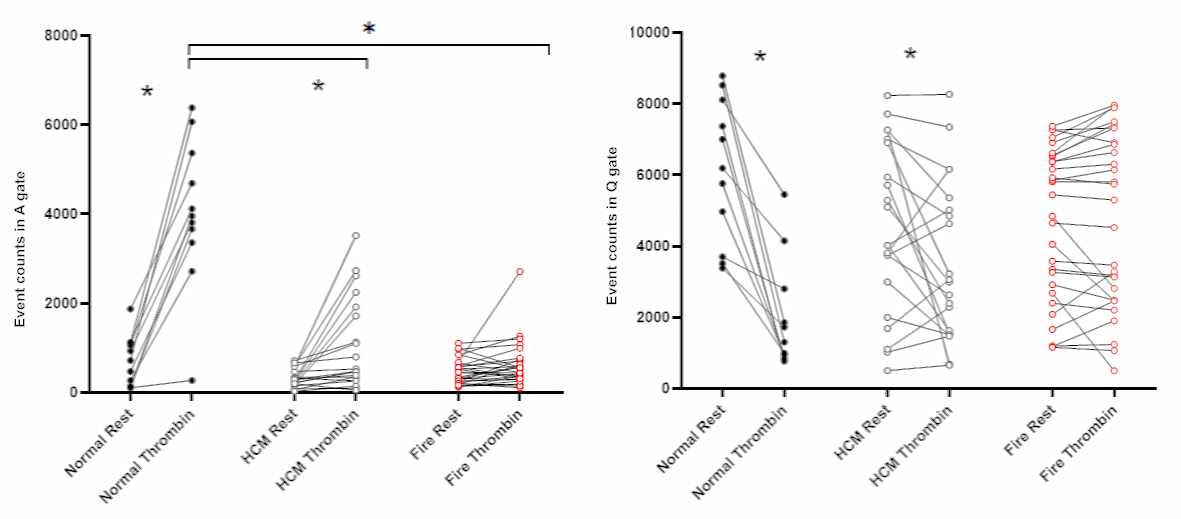

Supplement: Supplementary file 1 [file Image_1.tif]
